# Long-term follow-up of HCV infected kidney transplant recipients receiving direct-acting antiviral agents: a single-center experience in China

**DOI:** 10.1186/s12879-019-4217-7

**Published:** 2019-07-19

**Authors:** Jian Zhang, Wen Sun, Jun Lin, Ye Tian, Linlin Ma, Lei Zhang, Yichen Zhu, Wei Qiu

**Affiliations:** 1 Beijing Friendship Hospital, Capital Medical University, 95 Yongan Road, Xicheng District, Beijing, China; 2 Beijing key laboratory of Tolerance Induction and Organ Protection in Transplantation, Beijing, China

**Keywords:** Antiviral therapy, DAAs, HCV, Transplant

## Abstract

**Background:**

Long-term outcome of DAAs therapy in kidney transplant recipients was unknown. Thus, we aimed to evaluate it in a Chinese cohort of HCV-infected kidney transplant recipients.

**Methods:**

Single-center and retrospective study of HCV-infected kidney transplant recipients initiating an DAAs regimen between January 2015 and December 2017 was conducted. Totally 26 KTX recipients were divided into three groups, including KTX-HD Group, DAA-KTX Group and KTX-DAA Group. On-treatment response was defined as target not detected within 12 weeks. SVR 48, 96 were defined as HCV-RNA negativity 48, 96 weeks after treatment cessation, respectively.

**Results:**

HCV genotype was predominantly 1b (80.8%), followed by 2a. All (100%) patients achieved on-treatment response. Time to first TnD was 1.9 ± 0.6 weeks, with no significant difference among the three groups. All patients achieved SVR, with an SVR rate of 100.0% (26/26) among the patients who were followed up over 48 weeks after treatment cessation, and the same SVR rate (24/24) among the patients who were followed up over 96 weeks. Trough levels of Tac remained stable under DAAs therapy, without any dose adjustment. Two patients with abnormal GFR before treatment experienced serum creatinine elevation. Other adverse events included nausea, diarrhea, acid regurgitation, bilirubin elevation and edema of lower limbs. All patients recovered after treatment cessation without reductions in dose, or withdrawal of DAAs or immunosuppressive agents.

**Conclusions:**

HCV genotype 1b and 2a are the only genotypes and 1b is predominant in our center. Antiviral treatment with DAAs in HCV-infected kidney transplant recipients is persistently effective and well tolerated during long-term follow-up. A regular monitoring of renal function in patients who receive DAAs regimens with preexisting impaired renal function is strongly recommended. Furthermore, the trough CNIs levels were recommended to be frequently monitored.

## Summary of the article’s main point

Antiviral treatment with DAAs in HCV-infected kidney transplant recipients is persistently effective and well tolerated during long-term follow-up. A regular monitoring of renal function in patients who receive DAAs regimens with preexisting impaired renal function is strongly recommended. Furthermore, the trough CNIs levels were recommended to be frequently monitored.

## Background

Worldwide, approximately 118.9 million people are living with HCV RNA, indicating current or chronic infection, which corresponds to a global viraemic prevalence of 1.7% [1]. Furthermore, HCV infection is more prevalent among kidney transplant recipients than the general population [2], resulting in potential increased both morbidity and mortality [3]. High risk of HCV infection after a kidney transplant is thought to be, in part, secondary to the use of immunosuppressant, leading to the proliferation of the HCV.

Either poor efficacy or high prevalence of adverse effects have limited the use of pegylated interferon (IFN)-based regimens, especially for kidney transplant recipients, which may potentially trigger off an increased risk of allograft rejection and graft failure. However, the advent of direct-acting antiviral agents (DAAs), which are oral, IFN-free molecules that target HCV proteins, have taken the HCV treatment into a new era. Several studies demonstrated good outcome in achieving sustained virologic response (SVR) with minimal adverse effects in transplant recipients [2, 4–6], with the limitations of small sample size and short follow-up period. Thus, we aimed to evaluate the long-term outcome of DAAs therapy and the impact on plasma concentration of immunosuppressants in a Chinese cohort of HCV-infected kidney transplant recipients.

## Methods

### Patients

This study was conducted at Beijing Friendship Hospital, Capital Medical University (Beijing, China). We retrospectively reviewed all HCV-infected kidney transplant recipients that initiated an IFN-free DAAs regimen between January 2015 and December 2017. Totally 26 kidney transplant (KTX) recipients were enrolled, among whom 11 patients were on hemodialysis (HD) with renal allograft failure who received a low-dose Calcineurin inhibitor (CNIs)-based regimen (KTX-HD Group). Others with functioning renal allograft who received a higher-dose CNIs-based regimen than KTX-HD Group (KTX Group) were separated into two sub-groups, including DAAs treatment prior to transplant (DAA-KTX Group, *n* = 7) and after transplant (KTX-DAA Group, *n* = 8). Both HIV and HBV test were negative. None was treatment-experienced.

### Immunosuppressive regimens

Triple-therapy maintenance immunosuppression consisted of CNIs combined with both Mycophenolate mofetil (MMF) and prednisone (Pred). Double-therapy maintenance immunosuppression consisted of CNIs combined with either MMF or Pred. Monotherapy maintenance immunosuppression consisted of only CNIs. CNIs consisted of either tacrolimus (Tac) or cyclosporin A (CyA).

### HCV treatments

Genotype was confirmed and viral load was measured before therapy. The appropriate DAAs regimen was made according to annual guidelines. Except for one patient receiving 400 mg/QD sofosbuvir (SOF) monotherapy, others received 400 mg/QD SOF combined with either 90 mg/QD Ledipasvir (LDV) or 60 mg/QD Daclatasvir (DCV). Treatment response was evaluated by HCV-RNA at the first week, the second week, the fourth week, and every 12 weeks thereafter. On-treatment response was defined as target not detected (TnD) within 12 weeks. Sustained virologic response (SVR) 48, 96 were defined as HCV-RNA negativity 48, 96 weeks after treatment cessation, respectively. Adverse events were scored according to the Common Toxicity Criteria Adverse Events (CTCAE) version 4.0 [7].

### Statistical methods

Database management and statistical analysis were performed using SPSS 19.0. Continuous variables were expressed either as mean ± standard deviation for Gaussian distributions or median (range) for non-Gaussian distributions. Kolmogorov–Smirnov test was applied to determine whether continuous variables were normally distributed. Independent-sample t-test was used for statistical analysis. All statistical tests were two-tailed and *p* < 0.05 was considered statistically significant.

## Results

### Clinical characteristics

Overall, the HCV genotype was predominantly 1b (80.8%). Others were genotype 2a. Viral load before treatment was 6.5 ± 1.3 log_10_ IU/ml in total, with no significant differences among the three groups (5.9 ± 1.1 log_10_ IU/ml versus 6.5 ± 0.5 log_10_ IU/ml versus 7.2 ± 1.6 log_10_ IU/ml; *P*-value = 0.356, 0.448, 0.182). Except for one patient with cirrhosis receiving a 24-week therapy, others without cirrhosis received a standard 12-week DAAs therapy. At the initiation of DAAs therapy, 6 (75.0%) patients had a normal glomerular filtration rate (GFR) in KTX-DAA group, except for two patients with abnormal GFR of 45.1 and 27.0 ml/min, respectively. The characteristics of evaluated patients were given in Table 1.

**Table 1:** Characteristics of patients

Parameter	Total	KTX-HD	KTX	
			DAA-KTX	KTX-DAA
Patients	26	11	7	8
Age, years [mean ± SD]	49.0 ± 9.6	52.6 ± 10.0	39.7 ± 5.5	52.1 ± 6.6
Gender (male) [n (%)]	19 (73.1)	8 (72.7)	5 (71.4)	6 (75.0)
Transplant frequency [n (%)]				
1	12 (46.2)	6 (54.5)	2 (28.6)	4 (50.0)
2	12 (46.2)	5 (45.5)	4 (57.1)	3 (37.5)
≥3	2 (7.7)	0 (0.0)	1 (14.3)	1 (12.5)
HCV genotype [n (%)]				
1b	21 (80.8)	10 (90.9)	5 (71.4)	6 (75.0)
2a	5 (19.2)	1 (9.1)	2 (28.6)	2 (25.0)
Cirrhosis [n (%)]	1 (3.8)	0 (0.0)	0 (0.0)	1 (12.5)
Immunosuppressive regimens [n (%)]				
Triple-therapy	18 (69.2)	3 (27.3)	7 (100.0)	8 (100.0)
Double-therapy	6 (23.1)	6 (54.5)	0 (0.0)	0 (0.0)
Monotherapy	2 (7.7)	2 (18.2)	0 (0.0)	0 (0.0)
DAAs regimens [n (%)]				
SOF	1 (3.8)	0 (0.0)	1 (14.3)	0 (0.0)
SOF + LDV	17 (65.4)	8 (72.7)	4 (57.1)	5 (62.5)
SOF + DCV	8 (30.8)	3 (27.3)	2 (28.6)	3 (37.5)
Viral load, log_10_ IU/ml [mean ± SD]	6.5 ± 1.3	5.9 ± 1.1	6.5 ± 0.5	7.2 ± 1.6
Treatment cycle, weeks [median (range)]	12 (12-12)	12 (12-12)	12 (12-12)	12 (12-24)

### Virologic outcomes

All (100%) patients achieved on-treatment response. Time to first TnD was 1.9 ± 0.6 weeks, with no significant difference among the three groups (2.2 ± 0.6 weeks versus 1.7 ± 0.5 weeks versus 1.6 ± 0.5 weeks; *P*-value = 0.701, 0.453, 0.505). None experienced virologic relapse. All patients achieved SVR, with an SVR rate of 100.0% (26/26) among the patients who were followed up over 48 weeks after treatment cessation, and the same SVR rate (24/24) among the patients who were followed up over 96 weeks. The virologic outcomes were detailed in Table 2.

**Table 2:** Virologic outcomes

Parameter	Total	KTX-HD	KTX	
			DAA-KTX	KTX-DAA
On-treatment response [n (%)]	26 (100.0)	11 (100.0)	7 (100.0)	8 (100.0)
Time to first TnD, weeks [mean ± SD]	1.9 ± 0.6	2.2 ± 0.6	1.7 ± 0.5	1.6 ± 0.5
Virologic relapse [n (%)]	0 (0.0)	0 (0.0)	0 (0.0)	0 (0.0)
SVR 48 [n (%)]	26 (100.0)	11 (100.0)	7 (100.0)	8 (100.0)
Follow-up over 96 weeks after treatment cessation [n (%)]	24 (92.3)	9 (81.8)	7 (100.0)	8 (100.0)
SVR 96 [n (%)]	24 (100.0)	9 (100.0)	7 (100.0)	8 (100.0)

### Impact on plasma concentration of immunosuppressants

In KTX-DAA group, except for one patient receiving CyA-based therapy, others received Tac-based triple-therapy. Trough levels of Tac were randomly monitored before and after 4 weeks during treatment, which remained stable under DAAs therapy (3.8 ± 0.9 ng/ml versus 3.8 ± 1.4 ng/ml; *P*-value = 0.959), without any dose adjustment. The level of CyA was 95.4 ng/ml and 87.8 ng/ml before and after 4 weeks during the treatment, respectively, without any dose adjustment.

### Adverse events

**Fig. 1:**
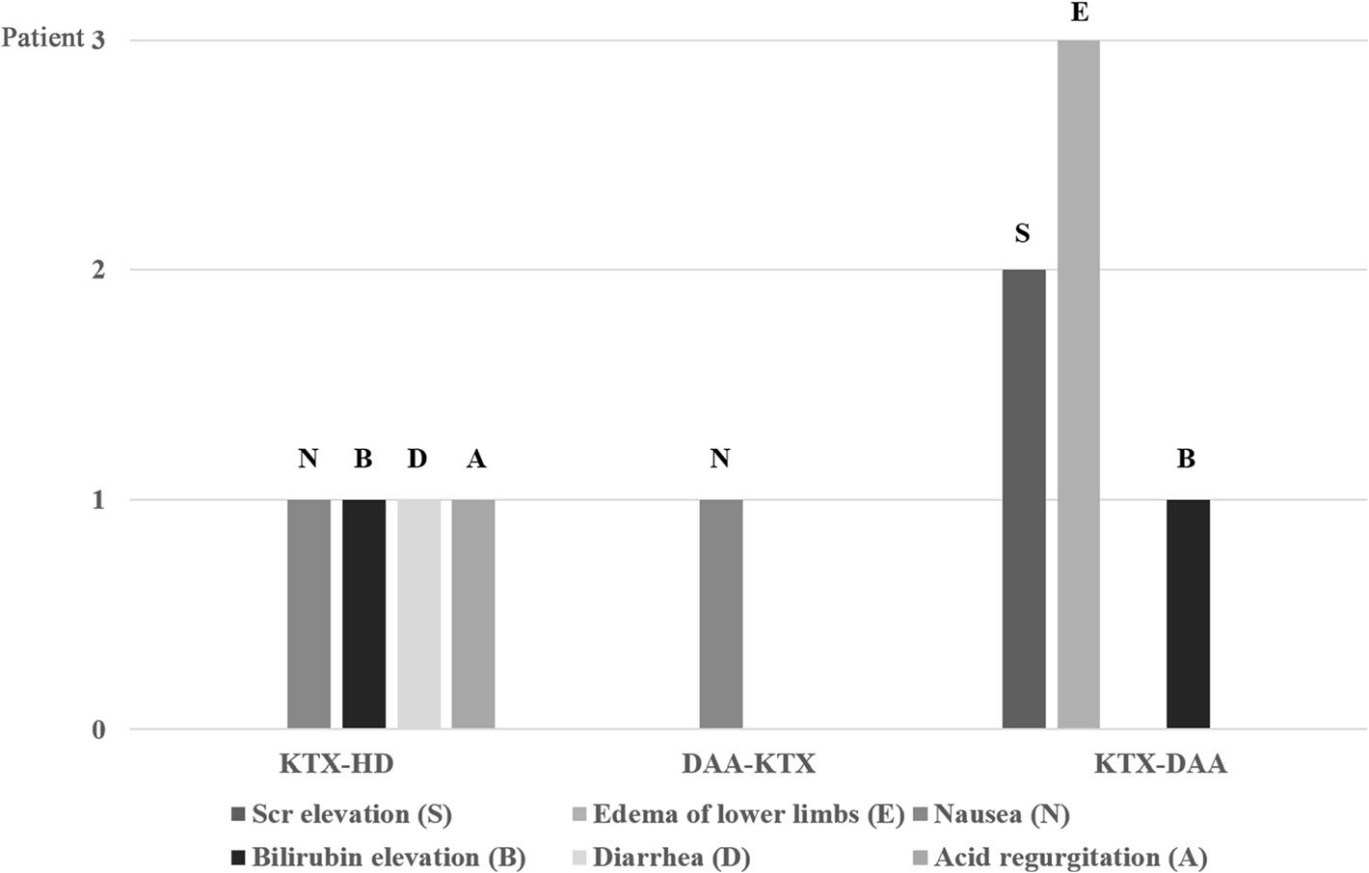
Adverse events of DAAs regimen

Adverse events (AEs) were observed in four (36.4%) patients in KTX-HD group, one patient (14.3%) in DAA-KTX group, and four (50.0%) patients in KTX-DAA group. Two patients with abnormal GFR before treatment experienced both serum creatinine (Scr) elevation (AE grade 1) and edema of lower limbs (AE grade 1). Except for one patient with bilirubin elevation (AE grade 2) in KTX-HD group, others experienced AEs grade 1. All AEs according to subgroups were given in Fig. 1. All patients recovered after treatment cessation without reductions in dose, or withdrawal of DAAs or immunosuppressive agents. None were hospitalized due to AEs.

## Discussion

According to the report from Chinese CDC, the estimated incidence of HCV was approximately 1.6 ‱ in 2017 [8]. A previous study indicated that genotype 1b and 2a were predominant in China, especially in the North [9]. Determination of HCV genotype was important for the choice of IFN-free DAAs regimens, that different HCV genotypes respond differently, until the emergence of pangenotypic DAAs [10]. However, pangenotypic DAAs were not in widespread use in China so that the prognosis was still highly associated with genotype. In our study, 1b and 2a were the only genotypes and 1b was predominant, which was consistent with previous study and considered to be curable by SOF-containing antiviral therapy.

The present study highlights the short-term efficacy of DAAs regimens among HCV-infected kidney transplant recipients, that the reported SVR 12 rate was 98.3%, ranging from 91.3 to 100.0% [11]. In our study, all patients achieved on-treatment response within four weeks, mostly two weeks, indicating both quick and effective response of DAAs regimens. The SVR rate of 100.0% in short-term follow-up was in accordance with previous studies [4, 12, 13]. In addition, the dose of CNIs seemed irrelative to the virologic response in this study. The latest study by Fernandez-Ruiz et al. also showed an SVR rate of 95.8% in the mid-term follow-up [14]. However, the long-term efficacy of DAAs has not been reported. Our study showed an SVR rate of 100.0% (26/26) among the patients who were followed up over 48 weeks after treatment cessation, and the same SVR rate (24/24) among the patients who were followed up over 96 weeks, demonstrating a good outcome in long-term follow-up.

The reported AEs included general symptoms (fatigue nausea dizziness or headache, 39.3%, 137/349), gastrointestinal symptoms (gastrointestinal bleeding or diarrhea, 7.2%, 25/349), and unstable blood pressure (1.1%, 4/349). The reported severe AEs included anemia, portal vein thrombosis, and streptococcus bacteraemia and pneumonia [11]. In our study, common AEs were partly different with the previous study. However, the association between DAAs and AEs was not clear. Nevertheless, none of severe AEs were observed and all patients recovered after treatment cessation, without reductions in dose, or withdrawal of DAAs or immunosuppressive agents, which was in accordance with the previous study, indicating the safety of DAAs regimens.

Chen et al. [11] reviewed six references and found no significant difference of Scr or GFR pre- and post-DAA therapy. However, a worsening proteinuria was observed in patients with proteinuria of greater than 500 mg/d before therapy by Lubetzky et al [5] A recent study also revealed a slight decrease in GFR during the first year following the end of treatment, while the graft function remained stable over the pre-treatment year [14]. In our study, Scr was not significantly changed in most patients, except for two patients with impaired but stable renal function before therapy, who had a Scr elevation (AE grade 1) without acute rejection and recovered after treatment cessation, that was consistent with the recent study by Brown et al. [15] Although doubtful, these data suggested possible association between AKI and SOF-containing DAAs administration. Furthermore, recent study on the histopathology showed acute interstitial nephritis in patients with AKI after SOF-containing antiviral therapy [16]. Thus, we recommend a regular monitoring of renal function in patients who receive DAAs regimens with preexisting impaired renal function.

Both Tac and CyA are substrates of liver cytochrome (CYP) P450 isoforms 3A4. Given the limited impact on CYP3A4 of SOF, LDV and DCV, no clinically relevant drug-to-drug interactions are expected when SOF, LDV or DCV coadministered with CyA or Tac [17, 18]. Lin et al. [19] reviewed 22 patients on CNIs treatment, of whom the majority had stable CNIs trough levels during antiviral treatment. The similar result was observed in recent study [20]. However, another study observed a significant decrease of trough Tac levels throughout the course of therapy, especially during the first month [14]. Bixby et al. [21] also found a great decrease of Tac concentration in the first 4 weeks of DAAs therapy and stable levels thereafter in a liver transplant cohort. Our study reviewed 7 patients with stable trough Tac levels before and after 4 weeks during DAAs therapy, without any dose adjustment, which revealed a different result from part of previous studies. However, due to the limit of sample and incomplete monitoring during the whole treatment, further study was required to verify this result. Given the risk of rejection, the trough CNIs levels were still recommended to be frequently monitored during DAAs therapy.

Several studies have demonstrated a significant survival benefit for HCV-infected patients receiving kidney transplant over remaining on dialysis [22]. Due to the increased risks of death, allograft loss and posttransplant complications for transplant with HCV, transplant clinicians have embraced pre-transplant treatment for HCV. However, with the development of DAAs, both necessity and superiority of pre-transplant treatment for HCV remains controversial. Unlike their uninfected counterparts, HCV-infected transplant candidates may have the option of being offered and accepting a kidney from an HCV positive donor, yielding the benefit of decreased time on the waitlist [13, 23]. In addition, our study found a good outcome of antiviral therapy after kidney transplant during long-term follow-up, weakening the necessity and superiority of pre-transplant treatment. Nevertheless, some clinical trials have focused on data collection of transplant outcomes allografts from HCV-infected donors to uninfected recipients [24], which might change the treatment strategies in the future.

Our study has several limitations. It is a retrospective study with a small sample size. All patients were treatment naïve and DAAs regimens were limited to SOF, LDV and DCV, so the results may not be generalizable to these other patient groups. Furthermore, the genotypes were limited to 1b and 2a, as would be expected in a study on a North Chinese population. Whether our findings would be applicable to other patient populations must be determined by larger prospective studies.

## Conclusions

HCV genotype 1b and 2a are the only genotypes and 1b is predominant in our center. Antiviral treatment with DAAs in HCV-infected kidney transplant recipients is persistently effective and well tolerated during long-term follow-up. A regular monitoring of renal function in patients who receive DAAs regimens with preexisting impaired renal function is strongly recommended. Furthermore, the trough CNIs levels were recommended to be frequently monitored.

## Data Availability

The datasets used and/or analysed during the current study are available from the corresponding author on reasonable request.

## Abbreviations

AEs: Adverse events
AKI: Acute kidney injury
CNIs: Calcineurin inhibitor
CTCAE: Common toxicity criteria adverse events
CyA: Cyclosporin A
CYP: Cytochrome
DAAs: Direct-acting antiviral agents
DCV: Daclatasvir
GFR: Glomerular filtration rate
HD: Hemodialysis
IFN: pegylated interferon
KTX: Kidney transplant
LDV: Ledipasvir
MMF: Mycophenolate mofetil
Pred: Prednisone
Scr: Serum creatinine
SD: Standard deviation
SOF: Sofosbuvir
SVR: Sustained virologic response
Tac: Tacrolimus
TnD: Target not detected

## References

[1] Petruzziello A, Marigliano S, Loquercio G, Cozzolino A, Cacciapuoti C (2016). Global epidemiology of hepatitis C virus infection: an up-date of the distribution and circulation of hepatitis C virus genotypes. World J Gastroenterol.

[2] Baid-Agrawal S, Pascual M, Moradpour D, Frei U, Tolkoff-Rubin N (2008). Hepatitis C virus infection in haemodialysis and kidney transplant patients. Rev Med Virol.

[3] Terrault NA, Adey DB (2007). The kidney transplant recipient with hepatitis C infection: pre- and posttransplantation treatment. Clin J Am Soc Nephrol.

[4] Goetsch MR, Tamhane A, Varshney M, Kapil A, Overton ET, Towns GC (2017). Direct-acting antivirals in kidney transplant patients: successful hepatitis C treatment and short-term reduction in urinary protein/creatinine ratios. Pathog Immun.

[5] Lubetzky M, Chun S, Joelson A, Coco M, Kamal L, Ajaimy M (2017). Safety and efficacy of treatment of hepatitis C in kidney transplant recipients with directly acting antiviral agents. Transplantation..

[6] Morales Amilcar L., Liriano-Ward Luz, Tierney Amber, Sang Michelle, Lalos Alexander, Hassan Mohamed, Nair Vinay, Schiano Thomas, Satoskar Rohit, Smith Coleman (2017). Ledipasvir/sofosbuvir is effective and well tolerated in postkidney transplant patients with chronic hepatitis C virus. Clinical Transplantation.

[7] National Institutes of Health National Cancer Institute. Common Terminology Criteria for Adverse Events (CTCAE) Version 4.0. Published: May 28, 2009 (v4.03: June 14, 2010).

[8] Chinese Center For Disease Control And Prevention. The epidemic of infectious diseases in China, 2017. Available at: http://www.nhc.gov.cn/jkj. Accessed 26 Feb 2018.

[9] Zhang Y, Chen LM, He M (2017). Hepatitis C virus in mainland China with an emphasis on genotype and subtype distribution. Virol J.

[10] European Association for the Study of the Liver (2018). EASL Recommendations on Treatment of Hepatitis C 2018. J Hepatol.

[11] Chen K, Lu P, Song R, Zhang J, Tao R, Wang Z (2017). Direct-acting antiviral agent efficacy and safety in renal transplant recipients with chronic hepatitis C virus infection: a PRISMA-compliant study. Medicine (Baltimore).

[12] Kamar N, Marion O, Rostaing L, Cointault O, Ribes D, Lavayssière L (2016). Efficacy and safety of Sofosbuvir-based antiviral therapy to treat hepatitis C virus infection after kidney transplantation. Am J Transplant.

[13] Sawinski D, Patel N, Appolo B, Bloom R (2017). Use of HCV+ donors does not affect HCV clearance with directly acting antiviral therapy but shortens the wait time to kidney transplantation. Transplantation..

[14] Fernández-Ruiz M, Polanco N, García-Santiago A, Muñoz R, Hernández AM, González E (2018). Impact of anti-HCV direct antiviral agents on graft function and immunosuppressive drug levels in kidney transplant recipients: a call to attention in the mid-term follow-up in a single-center cohort study. Transpl Int.

[15] Brown PR, Sadiq O, Weick A, Lenhart A, Elbatta M, Fernandez C (2018). Acute kidney injury in patients undergoing chronic hepatitis C virus treatment with Ledipasvir/Sofosbuvir. Hepatol Commun.

[16] Dashti-Khavidaki S, Khalili H, Nasiri-Toosi M (2018). Potential nephrotoxicity of sofosbuvir-based treatment in patients infected with hepatitis C virus: a review on incidence, type and risk factors. Expert Rev Clin Pharmacol.

[17] Burgess S, Partovi N, Yoshida EM, Erb SR, Azalgara VM, Hussaini T (2015). Drug interactions with direct-acting antivirals for hepatitis C: implications for HIV and transplant patients. Ann Pharmacother.

[18] Talavera Pons S, Boyer A, Lamblin G, Chennell P, Châtenet FT, Nicolas C (2017). Managing drug-drug interactions with new direct-acting antiviral agents in chronic hepatitis C. Br J Clin Pharmacol.

[19] Lin MV, Sise ME, Pavlakis M, Amundsen BM, Chute D, Rutherford AE (2016). Efficacy and safety of direct acting antivirals in kidney transplant recipients with chronic hepatitis C virus infection. PLoS One.

[20] Mansour M, Hill L, Kerr J (2018). Safety and effectiveness of direct acting antivirals for treatment of hepatitis C virus in patients with solid organ transplantation. Transpl Infect Dis.

[21] Bixby AL, Fitzgerald L, Leek R, Mellinger J, Sharma P, Tischer S (2019). Impact of direct-acting antivirals for hepatitis C virus therapy on tacrolimus dosing in liver transplant recipients. Transpl Infect Dis.

[22] Ingsathit A, Kamanamool N, Thakkinstian A, Sumethkul V (2013). Survival advantage of kidney transplantation over dialysis in patients with hepatitis C: a systematic review and meta-analysis. Transplantation..

[23] Shelton BA, Sawinski D, Mehta S, Reed RD, MacLennan PA, Locke JE (2018). Kidney transplantation and waitlist mortality rates among candidates registered as willing to accept a hepatitis C infected kidney. Transpl Infect Dis.

[24] Goldberg DS, Abt PL, Blumberg EA, Van Deerlin VM, Levine M, Reddy KR (2017). Trial of transplantation of HCV-infected kidneys into uninfected recipients. N Engl J Med.

